# Progressive Cerebral Atrophy and Cognitive Decline Without Cerebral Lesion in Neuromyelitis Optica Spectrum Disorder: A Case Report

**DOI:** 10.7759/cureus.97003

**Published:** 2025-11-16

**Authors:** Susumu Fujikawa, Fumitaka Shimizu, Masaya Honda, Michiaki Koga, Masayuki Nakamori

**Affiliations:** 1 Department of Neurology and Clinical Neuroscience, Yamaguchi University Graduate School of Medicine, Ube, JPN

**Keywords:** cerebral atrophy, cognitive decline, frontal lobe atrophy, neuromyelitis optica spectrum disorder, silent progression

## Abstract

Neurological deficits in neuromyelitis optica spectrum disorder (NMOSD) are conventionally thought to result from acute relapses, unlike multiple sclerosis (MS), which frequently progresses without relapse due to neurodegeneration. However, we present the case of a 41-year-old male patient who showed progressive bilateral frontal lobe atrophy over nine years, although acute relapses were limited to the medulla and spinal cord. Neuropsychological testing indicated cognitive decline. This case demonstrates progressive cerebral atrophy and cognitive decline, independent of relapses in NMOSD, possibly suggesting subclinical dying-back degeneration.

## Introduction

Neuromyelitis optica spectrum disorder (NMOSD), similar to multiple sclerosis (MS), is an inflammatory disease that causes recurrent lesions in the central nervous system. Their similarities had once led to a debate on whether NMO is the same as or different from MS [1] . However, NMOSD was established as a distinct disease entity different from MS, due to the discovery of aquaporin-4 (AQP4) antibodies in the IgG plasma fraction of patients with NMOSD [2] .

The clinical course of MS often presents "silent progression," which means progressive disability independent of relapses, resulting from neurodegeneration [3]. In patients with MS, silent progression is accompanied by brain atrophy and cognitive dysfunction [3,4]. In contrast to MS, it is generally conceived that, in the clinical course of NMOSD, silent progression is not observed, and all neurological impairment is caused by each relapse [5,6]. Therefore, neurodegeneration or brain atrophy is believed to be rare in patients with NMOSD. However, some reports have questioned this notion [7-9]. We herein report the case of a 41-year-old male patient with NMOSD who exhibited progressive brain atrophy over nine years, despite his young age and the absence of cerebral relapses. This case suggests neurodegeneration may progress independently of relapses in NMOSD.

## Case presentation

A 32-year-old male patient presented with intractable hiccups, nausea, and cough in 2011. He had no past medical history, no family history of neurological or neurodegenerative disorders, no regular medication use, no smoking, and only social alcohol consumption. He first visited the department of gastroenterology. Upper gastrointestinal endoscopy and abdominal CT were unremarkable, leading to suspicion of a psychogenic disorder.

**Figure 1:**
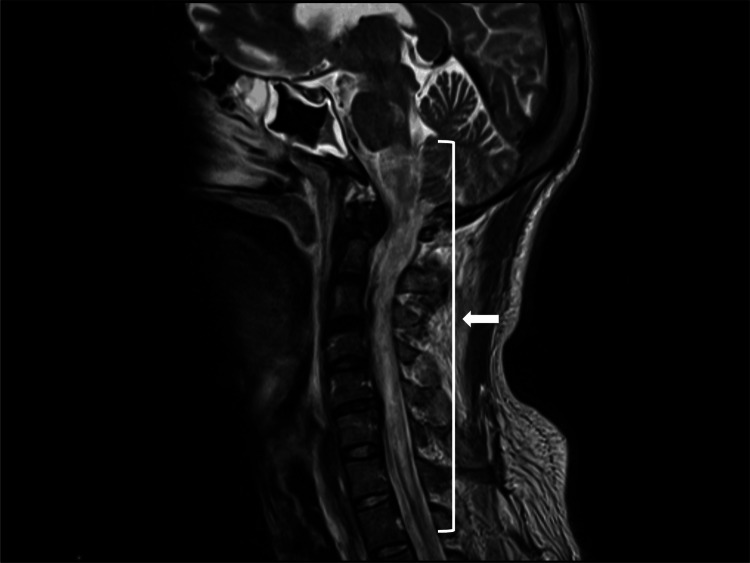
Initial T2-Weighted MRI Findings Sagittal T2-weighted MRI obtained at the first admission in 2011 showed a high-intensity lesion with swelling extending from the medulla oblongata to the Th1 level, indicated by the bracket and white arrow.

Five weeks later, nystagmus appeared, and after 10 weeks, he developed facial sensory disturbance, dysphagia, respiratory failure, and transverse myelopathy, including quadriparesis. MRI revealed longitudinally extensive transverse myelitis extending from the medulla to Th1 (Figure 1). The patient tested positive for serum AQP4 antibodies by a cell-based assay and was diagnosed with NMOSD. His dysphagia and respiratory failure required intubation and mechanical ventilation under ICU care during the acute phase. After treatment with steroid pulse therapy (methylprednisolone 1,000 mg/day for five days), 12 sessions of plasma exchange, and two courses of intravenous immunoglobulin therapy (0.4 g/kg/day for five days each), limb weakness, spasticity in the lower limbs, and urinary dysfunction persisted (the Expanded Disability Status Scale (EDSS) score was 7.5). Oral prednisolone therapy (15 mg/day) was initiated to prevent relapse.

Over the following years, the patient experienced two additional relapses, but his EDSS score remained essentially unchanged from the initial episode. In 2015, the patient relapsed with orthostatic hypotension due to a new medullary lesion while continuing the same dose of prednisolone. The symptoms improved after two courses of steroid pulse therapy (methylprednisolone 1,000 mg/day for three days each) and seven sessions of plasma exchange.

**Figure 2:**
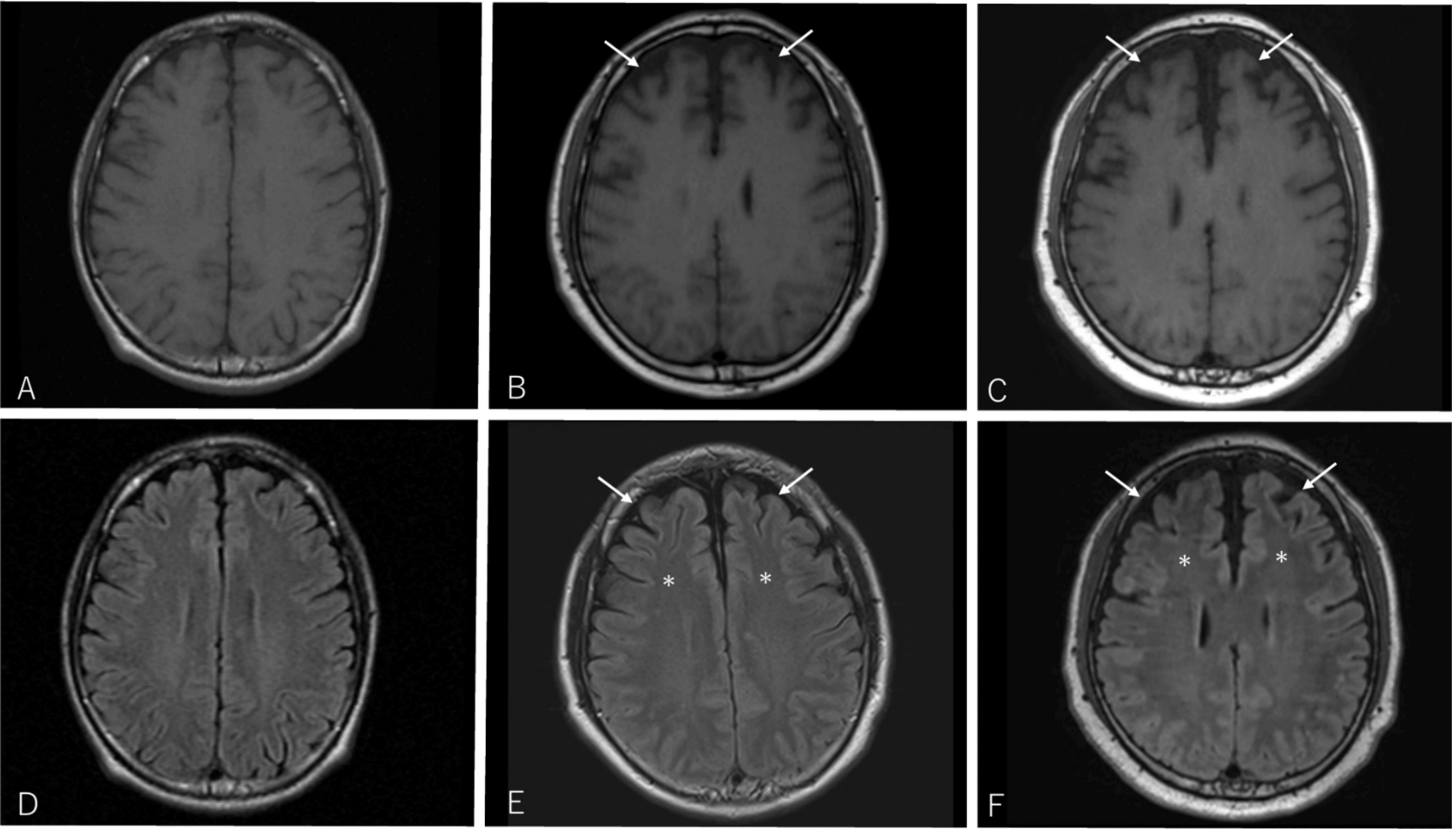
Serial Brain MRI Findings Over 9 Years Brain MRI of the patient in 2011 (A, D), 2015 (B, E), and 2020 (C, F). Serial T1-weighted (A–C) and FLAIR (D–F) images show progressive cortical atrophy in the bilateral frontal lobes over time. Arrows indicate areas of frontal cortical atrophy, and asterisks denote the absence of significant high-intensity lesions on FLAIR images. FLAIR: fluid-attenuated inversion recovery

In 2020, the patient was admitted to our department with intractable hiccups while taking prednisolone 12.5 mg/day. The patient exhibited persistent hiccups occurring every few seconds. Residual symptoms from the previous attacks included distal muscle weakness in both upper limbs, proximal muscle weakness in both lower limbs, spasticity and clonus in the lower limbs, hyperreflexia in the lower limbs, bilateral Babinski signs, and urinary dysfunction. The patient was diagnosed with a relapse of NMOSD because the brain MRI revealed a new enhanced lesion on the dorsal side of the medulla. The intractable hiccups disappeared after treatment with two courses of steroid pulse therapy (methylprednisolone 1,000 mg/day for three days each) and eight sessions of plasma exchange. Satralizumab was initiated as a relapse prevention therapy. Of note, the brain MRI performed in 2020 demonstrated progressive atrophy in the bilateral frontal lobes without cerebral white matter lesions, relative to that in 2011 (Figure 2), prompting neuropsychological testing.

The patient’s score on the Hasegawa Dementia Scale-Revised (Copyright © Ishiyaku Publishers, Inc.; used with permission for score summary only; this scale was selected as it does not require manual tasks because of hand motor impairment) [10] was 29 out of 30, indicating preserved global cognition on this dementia screening tool. However, more comprehensive testing revealed specific deficits. The Wechsler Adult Intelligence Scale, Fourth Edition (Copyright © 2008 NCS Pearson, Inc.; used with permission for score summary only) [11], revealed an overall IQ score of 76 (normal ≥85, borderline 70-85), indicating general cognitive decline. The perceptual reasoning score was 71, and the processing speed score was 71, reflecting specific deficits in these cognitive domains. Additionally, in the Wisconsin Card Sorting Test (Copyright © 1993 PAR, Inc.; used with permission for score summary only) [12], the patient achieved only 1 category (normal: 5-6), indicating impaired executive function.

Before disease onset, his cognition was suggested to be intact because he was able to work as a mid-level manager. After the disease onset, he discontinued working due to physical disability. Over the following years, obvious cognitive impairment was not evident in daily life. However, both his family and clinicians observed that he appeared somewhat slower and less efficient than before the illness. Throughout the clinical course, there was no history of exposure to benzodiazepines, other centrally acting medications, or substances known to potentially cause cerebral atrophy or cognitive decline. He had not consumed alcohol since the first onset in 2011. At the time of neuropsychological testing, neither significant fatigue nor depression was evident to influence performance. 

## Discussion

This report presents the case of a 41-year-old male patient with NMOSD who exhibited progressive brain atrophy and cognitive decline over nine years. It is believed that patients with NMOSD do not show neurological impairment with a progressive course without relapse, unlike MS, which exhibits silent progression without relapse [5,6]. However, recent reports have suggested progressive brain degeneration in patients with NMOSD. Similar to patients with MS, patients with NMOSD have significantly thinner cerebral cortices and lower cognitive function scores than healthy individuals [8]. Pathological findings have demonstrated the reduced expression of AQP4 in the superficial layers of the cerebral cortex and neuronal death in patients with NMOSD [7]. These changes are considered to reflect several NMOSD-related neurodegenerative processes, including AQP4-antibody-mediated excitotoxicity, microglial activation, meningeal inflammation, and potential autoimmune responses to other neural antigens [7].

Previous case reports have shown brain atrophy and cognitive dysfunction in three elderly patients with NMOSD who had cerebral lesions [13,14]. However, it is unclear whether brain atrophy and cognitive dysfunction in these cases were solely caused by the silent progression of NMOSD or were influenced by cerebral relapses, aging, and a concomitant primary neurodegenerative disorder, frontotemporal lobar degeneration (FTLD). In contrast, our patient was relatively young, had no cerebral relapses, and did not show overt dementia-level cognitive impairment. Rather, only a subtle decline was detected by detailed neuropsychological testing, making concomitant neurodegenerative disorders such as FTLD unlikely.

A retrospective cohort study investigated the longitudinal brain atrophy rate in 36 patients with NMOSD and 60 patients with MS, demonstrating similar silent progression of brain atrophy in both groups [9]. However, another cohort study comparing patients with NMOSD to healthy controls reported a higher longitudinal atrophy rate only in brain white matter, and this difference reached statistical significance only in cases with a history of cerebral syndrome associated with NMOSD [15]. This observation implies that the cerebral relapses contributed to brain white matter atrophy in that study. That is, whether brain atrophy can progress in NMOSD without cerebral relapses has not been demonstrated. In contrast, our report is the first to demonstrate progressive brain atrophy and cognitive decline in a patient with NMOSD without cerebral relapses.

Previous studies have shown that in spinal cord injury, dying-back degeneration originating from distal axonal disconnection leads to retrograde atrophy in the primary motor cortex (M1) and primary somatosensory cortex (S1) [16]. Moreover, transneuronal degeneration has been shown to result in progressive atrophy in the prefrontal cortex [17], which is connected to M1 via intermediate motor areas such as the premotor cortex and supplementary motor area [18]. In addition, patients with spinal cord injury often exhibit impairments in attention, processing speed, and executive function, with a tendency for these deficits to worsen over time [19]. In our case, the initial attack produced a markedly severe and extensive cord lesion. This lesion may have triggered a cascade of dying-back and transneuronal degeneration, extending from the spinal cord to M1 and further to prefrontal regions. This mechanism may have contributed, at least in part, to the progressive cerebral atrophy and cognitive decline observed in our patient.

## Conclusions

This case illustrates the possibility of progressive cerebral atrophy and cognitive decline in NMOSD, even in the absence of cerebral relapses. The present case is consistent with the notion that subclinical neurodegeneration may underlie NMOSD. These observations highlight the importance of long-term monitoring, including cognitive assessment, even in patients without cerebral relapses.
